# Review of Hand Reconstruction Methods: From Hand Pose to Neural Reconstruction of Hands with Category-Agnostic Objects

**DOI:** 10.3390/jimaging12090425

**Published:** 2026-09-09

**Authors:** Ahmed Elhayek

**Affiliations:** Artificial Intelligence Department, University of Prince Mugrin, Madinah 42381, Saudi Arabia; a.elhayek@upm.edu.sa

**Keywords:** hand pose estimation, hand shape modeling, two-hand interaction, hand–object reconstruction, category-agnostic, neural implicit representations, Gaussian splatting

## Abstract

Human hands play a central role in manipulation, communication, and physical interaction, making their accurate digital reconstruction a long-standing challenge in computer vision and graphics. Reliable modeling of hand pose, hand shape, and interaction is essential for applications ranging from immersive virtual and augmented reality to robotics, activity understanding, and human–machine interfaces. Over the past decade, research has evolved from isolated single-hand pose estimation toward increasingly holistic frameworks that jointly reconstruct two hands and the objects they manipulate. This review provides a comprehensive overview of hand reconstruction methods, tracing the progression from classical model-based approaches and early learning-driven pipelines to modern systems capable of two-hand interaction modeling and category-agnostic hand–object reconstruction. We structure the surveyed literature according to fundamental algorithmic paradigms, encompassing model-based formulations, convolutional and graph-based learning methods, transformer-based architectures, and emerging neural implicit and Gaussian representations. This review is tailored for researchers new to the field and follows a chronological and conceptual organization that elucidates how key design choices have shaped current capabilities and limitations. We analyze persistent challenges that hinder the widespread adoption of hand reconstruction methods in practical applications, including articulation complexity, generalization to unseen objects, and computational efficiency. Finally, this paper provides a forward-looking perspective on emerging research directions, highlighting trends toward real-time, category-agnostic, and semantically meaningful hand reconstruction systems for future human-centered computing.

## 1. Introduction

Human hands play a fundamental role in daily activities, serving as the primary means for manipulation, communication, and interaction with the surrounding environment. Consequently, understanding and reconstructing hand motion, pose, and shape have become central research topics in computer vision, robotics, and human–computer interaction [1]. Accurate hand reconstruction is critical for a wide range of applications, including activity recognition, immersive virtual environments, augmented and mixed reality (VR/AR/MR), teleoperation, digital content creation, and embodied artificial intelligence.

Early research efforts in this field primarily focused on *single-hand pose estimation*, aiming to recover the articulated skeletal configuration of a hand from visual observations [2,3]. While this line of work laid the foundation for hand analysis, modern applications demand richer representations. In particular, realistic 3D interaction scenarios require not only precise pose estimation but also *hand shape reconstruction* [4], *two-hand interaction modeling* [5,6], and explicit reasoning about *hand–object contact and manipulation* [7,8,9]. Despite substantial progress, hand reconstruction remains a challenging problem. The human hand exhibits a highly complex kinematic structure with many degrees of freedom, rapid motion, and frequent self-occlusions between fingers. Visual ambiguity caused by similar finger appearances, varying viewpoints, illumination changes, and background clutter further complicates robust estimation [10]. These challenges are amplified in scenarios involving two interacting hands or hands manipulating objects, where mutual occlusions and physical interaction constraints must be respected.

Advances in sensing technology and learning-based models have significantly reshaped the field. Depth sensors initially enabled more stable geometric reasoning [11], while deep learning later introduced powerful data-driven representations capable of handling complex appearance variations [12]. More recently, transformer-based architectures, neural implicit representations, and Gaussian-based scene representations have enabled joint reconstruction of hand pose, hand shape, and object geometry in unified frameworks [6,13,14]. Notably, category-agnostic hand–object reconstruction methods have emerged, allowing systems to generalize beyond predefined object classes and operate in unconstrained environments [8,9]. Given the rapid pace of development, new researchers entering the field often face difficulty navigating the diverse set of models, assumptions, input modalities, and output representations. Existing survey papers typically focus on narrow subproblems, such as single-hand pose estimation [15], RGB-only methods [16], or depth-based approaches [17], and often omit recent advances in two-hand interaction (such as [6,14]), hand–object reconstruction (e.g., [18]), and neural-rendering-based techniques [8,9].

This paper provides a comprehensive and accessible review of hand reconstruction research over the past decade, tracing the evolution of the field from early model-based single-hand pose estimation to modern methods capable of reconstructing two interacting hands and object pose and shape. The review is written with beginners in mind and follows a historical and conceptual progression. This paper provides the following contributions:

- A comprehensive and unified review of hand reconstruction research, spanning single-hand pose estimation, hand pose and shape modeling, two-hand interaction, and category-agnostic hand–object reconstruction.
- A structured taxonomy that categorizes existing methods according to core algorithmic paradigms, including model-based formulations, convolutional and graph-based learning, transformer architectures, and neural implicit and Gaussian-based representations.
- An integrated discussion of key enabling components, including datasets, hand models, input modalities, and output representations, highlighting their roles and interdependencies.
- A critical analysis of current limitations and open challenges, together with a forward-looking perspective on emerging research directions and future trends in hand reconstruction.

## 2. Related Surveys

Existing surveys have reviewed specific aspects of hand pose estimation. Early works predominantly focus on single-hand pose estimation in constrained settings, highlighting insights such as the effectiveness of volumetric 3D representations and the importance of structural constraints for handling occlusions [15,19,20]. Despite their value, these surveys remain narrowly centered on pose estimation and do not consider hand shape modeling or hand–object interaction. Some early reviews on articulated hand pose estimation categorize methods into discriminative, generative, and hybrid frameworks [17]. However, as these surveys predate recent learning-based developments, they offer a limited perspective and omit advances in hand shape estimation, two-hand interaction, and object-centric reconstruction. In contrast, recent progress in two-hand interaction modeling [6,14], hand–object reconstruction [18], and neural-rendering-based representations [8,9] has substantially expanded the scope of hand reconstruction. These emerging directions remain largely unaddressed by existing surveys.

Other surveys concentrate on specific sensing modalities, such as wearable-sensor-based hand pose estimation [21]. Although effective in controlled environments, these approaches require specialized hardware and exclude a wide range of practical scenarios where hardware-free, vision-based methods are now dominant. The survey by Supančič et al. [17] focuses on depth-based hand pose estimation methods, which generally achieve high accuracy because they provide explicit geometric information. However, these methods often fail in outdoor scenarios, where direct sunlight contains strong infrared (IR) components that interfere with active IR projection in most depth cameras, leading to severe noise, missing depth values, or complete sensor failure. In contrast, ref. [16] focuses on RGB-based hand pose estimation methods and analyzes progress despite the lack of depth cues. More broadly, both reviews provide structured analyses of both classical and learning-based methods, along with summaries of datasets available at the time. However, many of these surveys predate recent advances in large-scale datasets, modern deep learning architectures, and standardized benchmark protocols. Moreover, RGB-only reviews typically exclude depth and multimodal approaches, as well as hand shape modeling and hand–object interaction scenarios, which are essential components of contemporary hand reconstruction systems.

Consequently, there is still no unified review that systematically bridges single-hand pose estimation, two-hand interaction, hand shape modeling, category-agnostic object reconstruction, and neural rendering within a single framework. This paper fills this gap by presenting a concise, historically grounded, and task-complete overview of modern vision-based hand reconstruction methods. The review focuses on camera-based approaches that avoid wearable sensors, reflecting recent advances that have made purely visual systems both accurate and practical. Closely related but conceptually distinct problems—such as full human pose estimation—are therefore treated only peripherally; interested readers are referred to dedicated surveys for further details [22,23].

## 3. General Hand Reconstruction Concepts

**Figure 1:**
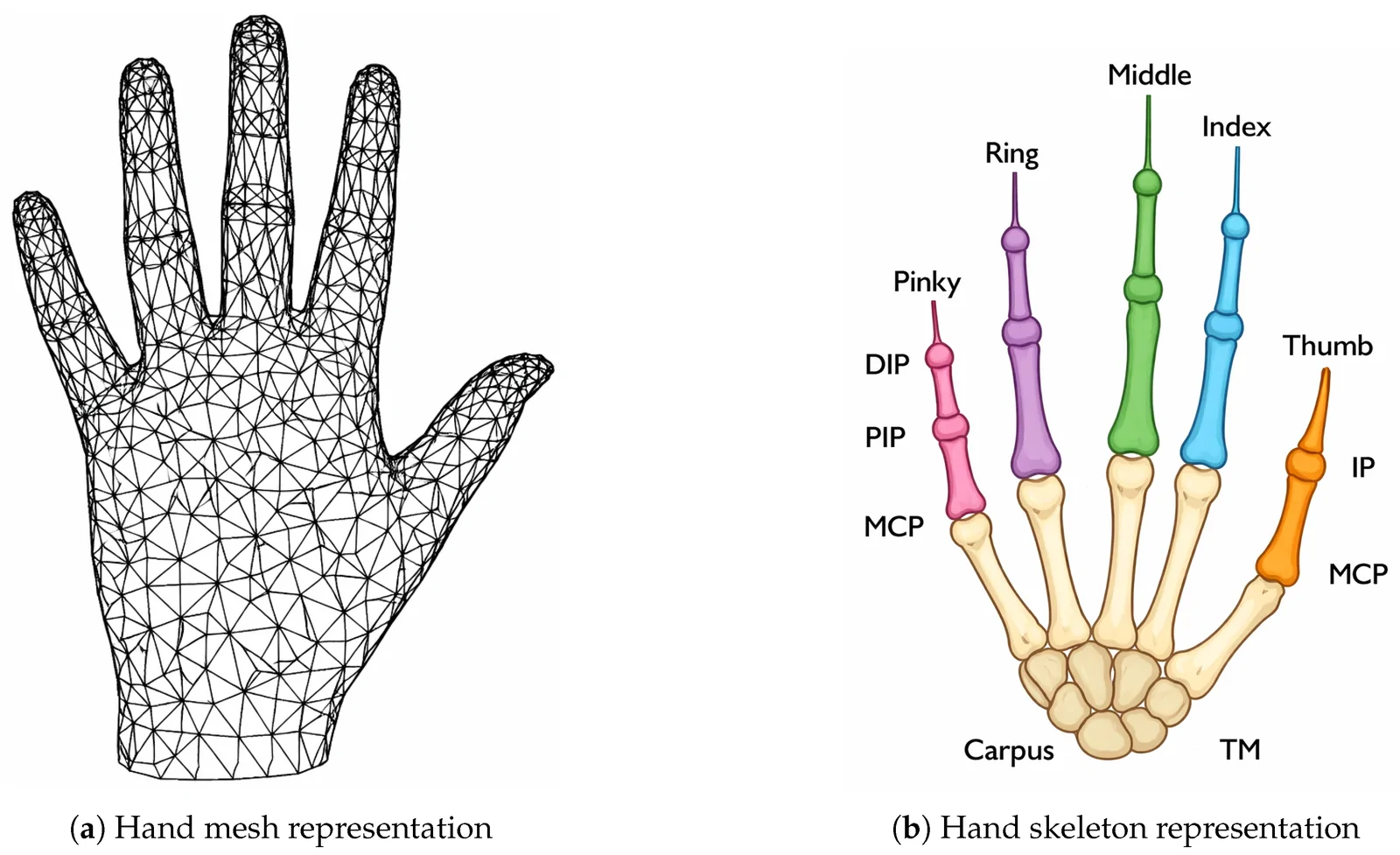
Comparison between hand mesh and skeletal representations used in hand reconstruction pipelines.

Before delving into the discussion of different hand reconstruction technologies, this section provides a brief background, as this paper targets readers who may be new to the field. Hand reconstruction methods generally aim to estimate either pose alone or both pose and shape. Accordingly, this section begins by defining these two conceptually distinct components to establish a clear foundation for the subsequent discussion. Figure 1b illustrates **Hand pose**, which refers to the spatial configuration of the hand skeleton, typically represented by a set of 2D or 3D joint locations corresponding to the palm and finger articulations. Pose estimation focuses on recovering the kinematic structure of the hand, including joint angles and relative positions, without explicitly modeling surface geometry. On the other hand, Figure 1a shows **Hand shape**, which describes the geometric surface of the hand, usually represented as a 3D mesh with a fixed topology (e.g., MANO model [24]). Hand shape captures anatomical characteristics such as finger thickness, palm width, and surface deformation, enabling realistic reconstruction and interaction modeling. While pose defines *how the hand moves*, shape defines *what the hand looks like*. Modern hand reconstruction systems often aim to estimate both pose and shape simultaneously, especially in scenarios involving interaction between two hands or between hands and objects.

**Figure 2:**
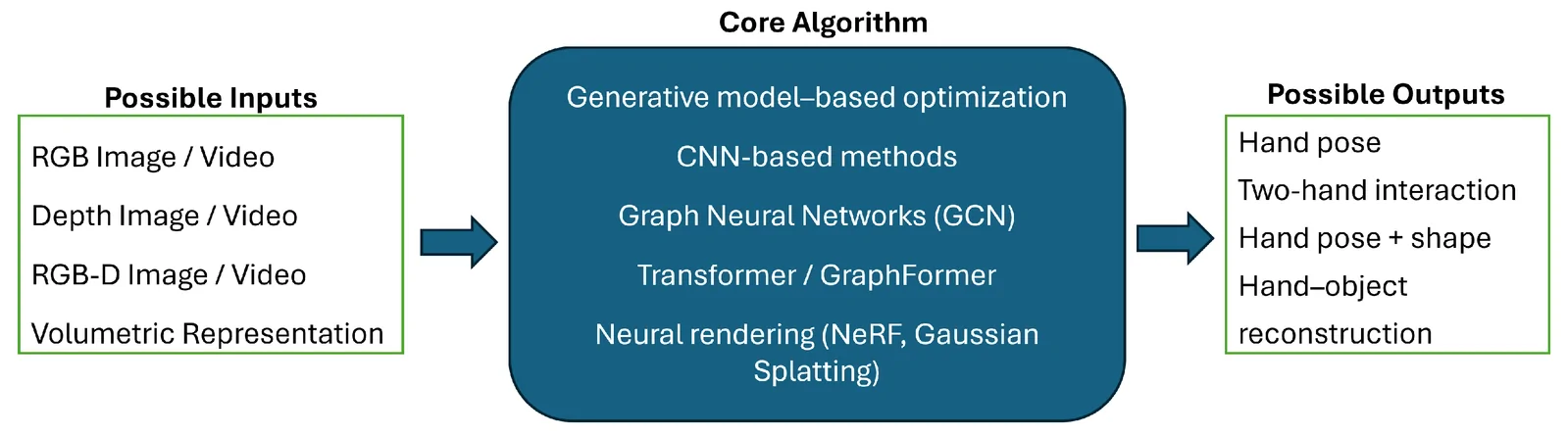
**General Hand Reconstruction Pipeline.** Overall pipeline of hand reconstruction systems, highlighting all possible *inputs*, *core algorithmic paradigms*, and *outputs*. The core processing stage comprises different algorithmic families.

Figure 2 presents a unified overview of a general hand reconstruction pipeline, illustrating the relationship between input modalities, core algorithmic components, and output representations.

### 3.1. Input Modalities

Hand reconstruction systems can operate on different types of input data depending on the sensing setup and application requirements:

- **RGB Images or Videos:** Monocular RGB images are the most widely used input modality due to their availability and suitability for numerous practical applications. However, these methods inherently suffer from depth ambiguity, making accurate 3D reconstruction challenging.
- **Depth Images or Videos:** Depth sensors provide explicit geometric information that significantly simplifies pose and shape recovery. However, despite their effectiveness in many scenarios, they may fail in some scenarios, such as outdoor environments where strong sunlight interferes with depth sensing, leading to noisy or missing measurements.
- **RGB-D Data:** The combination of color and depth improves robustness and accuracy, particularly for occlusions and complex hand articulations.
- **Volumetric Representations:** Some methods convert RGB-D data into voxel-based or implicit 3D representations, such as signed distance fields, to enable volumetric reasoning and neural rendering. This representation simplifies the learning process by allowing networks to operate directly in 3D space, reducing the ambiguity inherent in mapping from 2D images to 3D geometry and improving reconstruction accuracy, particularly for articulated hand models [25].

### 3.2. Core Algorithmic Paradigms

The central processing block of the pipeline represents the algorithmic backbone responsible for inferring hand pose and shape. Several major methodological paradigms have emerged in the literature to address this problem. In this section, this paper provides a brief overview of the basic concept of these approaches, while a more detailed discussion of each category is presented in the following sections.

- **Generative-Model-Based Optimization:** These methods fit parametric hand models (e.g., MANO) to image evidence using iterative optimization, often guided by reprojection or silhouette losses.
- **Convolutional Neural Networks (CNNs):** CNN-based approaches directly regress hand pose and shape parameters from input images by learning hierarchical feature representations. Some methods integrate task-specific layers within the network to explicitly convert latent pose and shape parameters into joint locations or mesh vertices, effectively embedding geometric reasoning into the architecture. By incorporating such structural layers, these models can more accurately enforce kinematic constraints and improve the consistency of predicted hand configurations [26].
- **Graph Convolutional Networks (GCNs):** Graph Convolutional Networks (GCNs) model the hand as a graph, where joints or mesh vertices are treated as nodes connected by anatomical edges. This representation preserves the inherent topology of hand pose and shape, enabling effective modeling of spatial and kinematic relationships. As illustrated in Figure 1, the graph structure naturally fits hand representations and allows GCN-based methods to handle complex interactions and occlusions more effectively [27].
- **Transformer-Based Models:** Transformers and GraphFormers capture long-range dependencies between joints or mesh vertices and have recently shown strong performance in hand pose estimation [18].
- **Neural Rendering Methods:** Techniques such as Neural Radiance Fields (NeRF) and Gaussian Splatting enable implicit 3D reconstruction and photorealistic rendering of hands, especially useful for view synthesis and category-agnostic object reconstruction.

### 3.3. Output Representations

Depending on the task definition and model design, hand reconstruction pipelines produce outputs at different levels of abstraction, ranging from single-hand pose estimation and two-hand interaction poses to full two-hand pose-and-shape reconstruction and joint hand–object pose-and-shape recovery. These output representations support a wide range of downstream applications, including virtual and augmented reality, human–computer interaction, robotic manipulation, and biomechanical analysis.

Figure 2 illustrates the modular nature of hand reconstruction pipelines, where different input modalities and algorithmic components can be combined depending on the application. Recent works have extended this paradigm toward category-agnostic hand–object reconstruction, where both the hand and previously unseen objects are reconstructed without relying on object-specific templates. For example, HOLD [9] demonstrates that implicit representations and interaction constraints can enable joint reconstruction of hands and unknown objects from monocular input, highlighting a shift from isolated pose estimation to holistic hand–object modeling.

## 4. Hand Reconstruction Technologies

This section reviews the fundamental technologies that have shaped hand reconstruction research over the past decade. The discussed methods are organized according to their underlying core algorithmic technology or learning paradigms. The section follows the chronological evolution of the field, tracing how technologies have progressed over time. For each stage, we highlight the key advances, as well as the advantages and limitations of the corresponding approaches, providing a clear perspective on how current methods have emerged.

### 4.1. Model-Based Methods

Model-based approaches represent one of the earliest and most influential paradigms in hand pose estimation. These methods explicitly encode hand kinematics and geometry, and estimate pose by fitting a parametric hand model to visual observations using optimization-based techniques. By enforcing anatomical and kinematic constraints, model-based approaches naturally restrict predictions to physically plausible configurations, making them particularly robust under noisy observations, partial occlusions, and limited training data.

While model-based methods are less prevalent in contemporary literature because of the shift toward data-driven deep learning, they remain fundamentally important for understanding the core principles of hand pose estimation and continue to inspire new research directions. Moreover, since these methods are not data-driven, they do not rely on large annotated datasets and are therefore less sensitive to variations and biases in training data. A comprehensive comparison of model-based techniques is presented in [15], which analyzes different strategies for hand model construction and tracking. The same work introduces sphere-meshes as a geometric representation for real-time generative hand tracking, demonstrating that tracking accuracy is highly sensitive to how closely the model matches the specific user’s hand shape. Similarly, Sridhar et al. [28] propose a generative hand tracking method based on an implicit hand shape representation using a Sum of Anisotropic Gaussians (SAG). Their formulation employs a smooth and analytically differentiable pose fitting energy, enabling efficient and accurate optimization. In the remainder of this section, this paper provides a more detailed discussion of generative (i.e., model-based) hand pose estimation methods.

#### 4.1.1. Classic Optimization and Kinematic Fitting

Early work in hand reconstruction relied on generative formulations that optimize a predefined hand model to match image evidence such as silhouettes [29], depth maps [30], or detected keypoints. The hand is typically represented as an articulated skeleton with a fixed kinematic tree, and pose estimation is formulated as minimizing an energy function subject to joint-angle constraints [31,32,33]. These methods produce interpretable and physically valid results, but often suffer from high computational cost, sensitivity to initialization, and susceptibility to local minima—especially in the presence of fast motion or strong self-occlusions.

#### 4.1.2. Strengths and Limitations

Model-based approaches offer several advantages, including enforced physical plausibility through explicit kinematic constraints, strong interpretability due to structured parametric representations, and robustness in scenarios with limited training data. However, these methods also exhibit notable limitations, such as susceptibility to local minima during optimization, high sensitivity to initialization, and a limited capacity to accurately model soft-tissue deformation and subject-specific shape variation. Consequently, while model-based priors remain highly valuable, modern hand pose estimation systems increasingly integrate them with data-driven components—such as graph neural networks, transformers, and implicit or Gaussian representations—to improve generalization and achieve more realistic modeling in complex interaction scenarios.

In summary, model-based methods form the conceptual foundation of hand reconstruction research. Although purely generative approaches are gradually being replaced by hybrid and learning-based models, explicit kinematic constraints continue to play a crucial role in ensuring physically consistent and interpretable predictions—particularly in scenarios involving limited supervision.

### 4.2. CNN-Based Methods

Convolutional Neural Networks (CNNs) have become the dominant paradigm for hand pose and shape estimation due to their strong representation capacity and ability to learn complex mappings directly from visual data. CNN-based approaches typically regress hand pose and/or shape from depth, RGB, or RGB-D inputs using layered convolutional architectures, significantly outperforming earlier handcrafted and decision-forest-based methods. This subsection reviews the evolution of CNN-based hand pose and shape estimation methods, with a focus on datasets, early pose-only models, hybrid formulations, and volumetric representations.

#### 4.2.1. Datasets Enabling CNN-Based Learning

The success of CNN-based methods is closely tied to the availability of large-scale annotated datasets. Early progress was enabled by real-world benchmarks such as the NYU Hand Pose Dataset [2], which provides depth images annotated with 3D joint locations using a model-based fitting procedure. Despite covering a wide range of poses, NYU is limited to a single hand shape, restricting shape generalization. To address data scale limitations, Yuan et al. introduced the BigHand2.2M dataset [34], which contains over two million depth images with accurate joint annotations and large pose diversity. However, this dataset still exhibits limited hand shape variation, as it includes only a small number of subjects. These datasets collectively made large-scale supervised training of CNNs feasible, establishing the foundation for modern hand pose and shape estimation methods.

#### 4.2.2. Early CNN-Based Methods

The earliest CNN-based approaches focused solely on estimating 3D hand joint locations. These methods typically employ standard 2D CNN architectures to directly regress joint coordinates or predict per-joint heatmaps from depth images. One of the pioneering works by Tompson et al. [2] combined CNN-based dense feature extraction with a subsequent inverse kinematics step to recover stable 3D hand poses in real time. Following this direction, several discriminative approaches directly regressed 3D joint positions using CNNs, achieving significant improvements over traditional methods. Although highly accurate, these early CNN-based pose-only models generally lacked explicit geometric or kinematic constraints, which could lead to physically implausible predictions under severe occlusion or noise.

#### 4.2.3. Hybrid CNN-Based Methods with Embedded Hand Models

To incorporate physical structure into learning-based systems, hybrid approaches emerged that embed hand model layers within CNN architectures. These methods combine the representational power of CNNs with explicit hand kinematics or shape models. A representative example is the integration of forward kinematics layers into deep networks [35], allowing CNNs to predict joint angles that are subsequently converted into 3D joint positions through a differentiable kinematic model. Extensions of this idea further allow the estimation of hand shape parameters, enabling the reconstruction of full hand meshes from learned latent representations [24,36]. Such hybrid formulations improve physical plausibility and interpretability, while maintaining the accuracy benefits of CNNs. However, their performance is often constrained by the expressiveness of the underlying hand model and the limited shape variation encoded in parametric representations.

#### 4.2.4. Volumetric CNN-Based Representations

To better capture the inherently three-dimensional nature of depth data, volumetric CNN-based methods have been proposed. Instead of treating depth maps as 2D images, these approaches convert inputs into 3D voxel grids, enabling the use of 3D convolutions to learn spatially consistent representations. The voxel-to-voxel framework introduced by Moon et al. [37] establishes a one-to-one mapping between voxelized depth inputs and voxelized 3D joint heatmaps, leading to highly accurate pose estimation. Building upon this idea, subsequent works extended volumetric representations to hand shape estimation, predicting full 3D occupancy grids or implicit surfaces from voxelized inputs [25]. Volumetric methods offer superior spatial reasoning and naturally preserve 3D structure, but they come with increased computational and memory costs. Despite these challenges, volumetric CNNs represent one of the most effective paradigms for joint hand pose and shape estimation from depth data.

In summary, CNN-based hand pose and shape estimation has evolved from simple pose regression models to structured hybrid systems and fully volumetric architectures. Each progression reflects a trade-off between accuracy, physical plausibility, and computational complexity, motivating ongoing research toward unified and efficient representations.

### 4.3. Graph Convolutional Networks Methods

Hand pose and shape estimation naturally involves *structured outputs*. The hand skeleton can be modeled as a kinematic graph in which nodes correspond to joints and edges encode anatomical connections, while the hand surface is typically represented as a triangular mesh—a graph whose nodes are mesh vertices and whose edges reflect surface connectivity. Similarly, object geometry is often represented using a mesh or a deformable mesh template, making graph-based learning a natural and unified formulation for both hand and object reconstruction. Graph Convolutional Networks (GCNs) exploit this structure through *message passing*, where information is iteratively aggregated from local neighborhoods to learn topology-aware representations. This property is particularly beneficial for (i) enforcing local geometric consistency on dense meshes, (ii) propagating kinematic constraints along articulated chains to ensure physically plausible poses, and (iii) refining noisy predictions under occlusion or missing depth, which are common in interaction scenarios.

Importantly, GCNs are highly flexible with respect to their input features: each graph node can be associated with an arbitrary vector embedding, extracted from RGB images, depth maps, or intermediate 3D representations such as point clouds or voxel grids. As a result, GCN-based pipelines only require an encoder that maps the raw input data into per-node feature vectors, which are then embedded on a predefined graph structure—such as a rest-pose hand skeleton, a template hand mesh, or an object mesh—enabling seamless integration of appearance and geometry within a unified graph reasoning framework.

#### 4.3.1. Datasets Enabling Graph-Based Learning in Interaction Settings

As with CNN-based approaches, the success of GCN-based reconstruction is strongly tied to the availability of datasets that provide reliable 3D supervision for *both* hands and objects, particularly in the presence of mutual occlusions. Datasets focusing on isolated hands, such as NYU [2] and BigHand2.2M [34], have been discussed earlier. Here, we focus on datasets that explicitly capture hand–object manipulation scenarios [38], which are crucial for enabling topology-aware learning and joint reconstruction. HO-3D [39] is a widely adopted benchmark that provides aligned RGB (and, in some settings, depth) data together with annotations for both hand pose and object pose across interaction sequences. It has become a standard evaluation platform for learning-based hand–object reconstruction. DexYCB [13] further increases the diversity of manipulation scenes and object categories, offering larger-scale supervision and improved variability compared to earlier interaction datasets. Additional resources such as GRAB [40] and contact-focused datasets, including ContactPose and ContactDB [41,42], have further supported research on physically meaningful interaction cues, such as contact regions. These datasets are particularly well suited for graph-based formulations, as contacts can be naturally modeled as constraints or edges between nodes on the hand and object surfaces. Collectively, these benchmarks have enabled a shift from isolated hand reconstruction toward realistic manipulation settings characterized by severe occlusions and complex surface-to-surface interactions [13,39,40,41,42].

#### 4.3.2. Pure Hand Reconstruction

A common and effective design is to use a CNN/voxel encoder to extract global visual features, followed by a GCN that decodes or refines a mesh in a topology-aware manner. This division of labor is well-motivated: pixel/voxel encoders capture appearance and global context, while GCNs enforce local coherence by propagating information along mesh connectivity, reducing artifacts that often arise when vertices are regressed independently. Dense hand mesh recovery has benefited from graph-based decoding and refinement, especially in monocular settings where mesh reconstruction is underconstrained. For example, graph reasoning has been used to predict or refine vertex-level deformations on a fixed-topology hand mesh, improving surface smoothness and preserving anatomical structure compared to direct vertex regression baselines [24,43,44]. In depth-based pipelines, voxel-centric encoders provide strong geometric cues, and subsequent topology-aware refinement further improves mesh consistency from partial observations. HandVoxNet-style pipelines highlight the utility of operating in the 3D domain (voxelized depth) and complementing volumetric reasoning with structured refinement for more stable and coherent surface recovery [25,45]. More broadly, graph-based mesh reconstruction paradigms developed for humans have informed hand mesh recovery as well, especially where a fixed template topology is refined via learned vertex offsets under neighborhood regularization [24,46].

#### 4.3.3. Joint Hand–Object Reconstruction

Graph reasoning becomes even more valuable when reconstructing hands interacting with objects, where *mutual occlusion* and *physical constraints* (contact, non-penetration) introduce additional ambiguity. Joint hand–object reconstruction methods often either (i) estimate hand and object separately and then enforce interaction constraints, or (ii) jointly predict both geometries in a coupled manner. In both cases, GCNs provide a principled mechanism to couple information across structured domains: within the hand mesh, within the object mesh, and across hand–object contact neighborhoods. Interaction-focused pipelines often build on strong monocular baselines that estimate hand pose/shape and object pose from RGB images [7,27,47,48,49], and then incorporate additional mechanisms to reason about hand–object consistency. Classical optimization-based joint tracking from RGB-D demonstrated the importance of physical plausibility and interaction constraints, but required known object geometry or costly per-sequence fitting [50,51,52]. More recent learning-based approaches increasingly adopt graph-based components to reconstruct or refine interacting surfaces, either by improving dense geometry recovery of the hand, or by co-regularizing hand and object shape predictions in contact-rich regions [18,45]. The availability of HO-3D and DexYCB has been essential here, enabling supervision for both hand and object and making it feasible to learn interaction-aware shape predictors that remain robust under heavy occlusion [13,39]. Another recent graph-based methods such as HHMR improve robustness under occlusion [53]. HHMR combines graph diffusion with graph convolution and attention so the model can better propagate global context and recover plausible hand meshes from ambiguous inputs.

In summary, GCNs are well-matched to hand and object reconstruction because both skeletal pose and surface geometry are naturally graph-structured. By operating directly on skeleton graphs and mesh graphs, GCN-based methods provide topology-aware refinement and dense surface consistency, and they integrate naturally into interaction pipelines where reasoning over contact neighborhoods and mutual occlusions is critical [13,18,25,39,45].

### 4.4. Transformer-Based Methods

Transformer architectures have become a cornerstone in modern vision pipelines due to their ability to model *global context* and *long-range dependencies* via self-attention [54]. Originally developed for sequence modeling, transformers have demonstrated strong performance across a wide range of computer vision tasks, motivating the adoption of Vision Transformers (ViTs) for dense, structured prediction problems [55]. In hand reconstruction, attention-based reasoning is particularly appealing because hands frequently exhibit self-occlusion, fine-grained articulation, and ambiguous appearance; transformers can aggregate evidence across distant regions and capture part-to-part dependencies beyond local receptive fields typical of CNNs. Consequently, transformer backbones and decoders are increasingly used to regress hand pose, MANO parameters, and dense meshes, often yielding improved robustness under occlusion and in-the-wild variability [56,57,58].

#### 4.4.1. Datasets and Scale Requirements

A key practical challenge for transformer-based methods is their data hunger: in contrast to CNNs, which often train reasonably well on moderate-scale datasets, transformers typically require substantially larger and more diverse supervision to generalize reliably [55]. For hand pose and mesh recovery, early datasets alone (e.g., NYU [2], BigHand2.2M [34]) are insufficient to fully exploit transformer capacity, particularly for in-the-wild generalization. Recent state-of-the-art pipelines therefore rely on *large-scale unified training corpora* assembled from multiple sources that provide complementary supervision signals. A representative example is HaMeR [57], which is trained on approximately 2.7M annotated samples aggregated from diverse datasets with both 2D and 3D supervision. This combined corpus includes datasets with 3D annotations such as FreiHAND [59], HO3D [39], H2O3D [39], InterHand2.6M [60], MTC [61], DexYCB [13], and the synthetic RHD [62], as well as large-scale 2D-supervised sources such as COCO WholeBody [63], Haple-FullBody [64], and MPII+NZSL [65]. Unifying these heterogeneous datasets into a consistent representation is essential for effective large-scale training and has become a critical enabler for transformer-based hand reconstruction [57,58]. In interaction-centric settings, HO3D and DexYCB in particular provide challenging hand–object manipulation scenarios with severe mutual occlusions, and thus serve as standard benchmarks for evaluating generalization under contact-rich conditions [13,39].

#### 4.4.2. Pure Transformer-Based Methods

Pure transformer pipelines typically follow an “encode–decode” design: a ViT or transformer-based encoder produces global image tokens, and a transformer decoder maps these tokens to structured hand outputs (e.g., joints, mesh vertices, or MANO parameters). Such designs benefit from direct token-to-token interactions, which facilitate reasoning across distant hand parts and can improve robustness when large regions are occluded. HaMeR [57] exemplifies this family by combining a ViT-H backbone [55] with a transformer decoder to regress MANO [24] parameters, achieving strong performance on in-the-wild imagery through large-scale training. Similarly, HandOccNet [56] leverages attention-driven reasoning to better handle occlusion by exploiting correlations between visible and occluded regions. Beyond hands, transformer decoders have been shown effective for dense mesh recovery in related settings (e.g., human mesh recovery), which has influenced hand reconstruction design choices; for instance, METRO [66] demonstrates how transformer encoders can jointly model vertex–vertex and vertex–joint interactions through self-attention [54]. These ideas transfer naturally to hands, where structured prediction involves similarly dense and correlated outputs. Despite their accuracy, large transformer backbones are computationally expensive, increasing memory footprint and inference latency, which can limit real-time deployment on resource-constrained devices [57,58]. This practical constraint has motivated research into more efficient transformer variants and training strategies, including lightweight backbones, model compression, and knowledge transfer, without sacrificing reconstruction quality [67].

#### 4.4.3. GraphFormers Methods

While transformers provide strong global reasoning, they do not explicitly encode mesh or kinematic topology. Graphformer approaches address this by combining self-attention with graph-based operators, enabling both *global token interactions* and *topology-aware local propagation*. This hybridization is particularly relevant for hand meshes, where fixed connectivity provides a strong prior for spatial smoothness and anatomical plausibility. Mesh Graphormer [68] is a representative hybrid design that integrates self-attention [54] with graph convolutions to better exploit mesh structure during dense reconstruction. Similarly, GraFormer-style formulations [69] combine transformer reasoning with graph message passing, strengthening global coherence while preserving local topological consistency. In interaction settings, hybrid transformer–graph designs have been adopted to jointly reason about multiple entities under mutual occlusions. For example, THOR-Net [18] combines attention-driven reasoning with graph-based modeling and self-supervision to estimate the pose and shape of two hands interacting with an object, highlighting the benefit of coupling global context with topology-aware refinement for manipulation scenarios.

Recently, HandDAGT, MaskHand, and HORT were introduced [70,71,72]. HandDAGT uses an adaptive graph-transformer design to model hand-joint topology and improve 3D hand pose estimation under occlusion [70]. MaskHand introduces a context-guided masked-transformer framework that improves in-the-wild generalization for 3D hand mesh reconstruction [71]. HORT applies conditional transformers for human-centric reconstruction of hand-held objects while jointly reasoning about hand–object structure [72].

In summary, Transformer-based methods have shifted hand reconstruction from predominantly local feature regression to global, attention-driven reasoning, achieving strong robustness under occlusions and in-the-wild variability when trained at scale [56,57,58]. Their success relies heavily on large, unified datasets that combine 2D and 3D supervision across diverse conditions [13,39,59,60,63,64]. Graphformer methods further enhance transformer pipelines by incorporating explicit topology through graph operations, improving dense mesh recovery and interaction reasoning in contact-rich settings [18,68,69].

### 4.5. Neural Implicit and Gaussian-Based Methods

Recent advances in hand and hand–object reconstruction have been strongly driven by *neural implicit representations* and their more recent explicit counterparts based on Gaussian Splatting. Unlike mesh-centric or template-based pipelines, which rely on fixed topology or a limited set of predefined object models, neural and Gaussian-based formulations represent geometry and appearance in a continuous or point-based manner. This flexibility enables *category-agnostic object reconstruction*, allowing systems to generalize to unseen and unconventional object shapes encountered in real-world interaction scenarios.

Despite substantial progress, existing hand–object reconstruction methods largely fall into two categories. Template-based approaches [7,73,74] can generate high-quality and physically consistent meshes, but are inherently restricted to a small, fixed set of known object templates, limiting their ability to generalize. Category-agnostic methods remove this constraint, but typically incur high computational cost and optimization complexity [8,9], and often struggle to recover missing geometry or realistic hand–object contact under severe occlusions [18].

#### 4.5.1. Data Requirements and Sequence-Based Supervision

Neural implicit and Gaussian-based representations differ fundamentally from discriminative mesh regression methods in their data requirements. Rather than relying on large collections of independent images, these approaches typically require *multi-view or temporal sequences* to accumulate sufficient geometric and photometric constraints. Consequently, progress in this area has been enabled by datasets that provide synchronized video sequences with camera motion, hand pose estimates, and interaction diversity. Benchmarks such as ARCTIC Bi-CAIR [9,75] provide bimanual hand–object interaction sequences with diverse grasp types and severe occlusions, making them well suited for category-agnostic reconstruction. HO3D [39], discussed earlier, is suitable for this type of method because it offers controlled interaction scenarios with accurate annotations. Thus, it is commonly used to evaluate robustness under hand-induced occlusion. Compared to static image datasets, such sequence-based benchmarks are essential for implicit and Gaussian pipelines, as they enable multi-view consistency, temporal regularization, and stable geometry recovery over time.

#### 4.5.2. Neural Implicit Fields

Neural implicit representations, most notably Neural Radiance Fields (NeRFs) [76], model scenes as continuous functions that map 3D coordinates and viewing directions to radiance and density. This paradigm has enabled photorealistic novel-view synthesis and high-fidelity geometry recovery, and has been adopted for hand-centric and interaction-aware reconstruction [77]. In the context of category-agnostic hand–object reconstruction, HOLD [9] represents a canonical NeRF-based pipeline. It combines off-the-shelf components for hand pose estimation [57,66], object segmentation [78,79], and camera pose estimation [80,81,82], followed by per-sequence NeRF optimization to jointly recover object geometry and appearance from monocular RGB videos. While such pipelines can achieve strong photorealism, they typically require hours of optimization per sequence [9], making them unsuitable for interactive or real-time applications.

#### 4.5.3. Gaussian Splatting as an Explicit and Efficient Alternative

Gaussian Splatting [83] offers an explicit 3D representation in which scenes are modeled as collections of 3D Gaussians with learnable attributes such as position, covariance, opacity, and appearance features. Rendering is performed by projecting and blending Gaussians in image space, enabling efficient and stable differentiable rendering. Compared to NeRF-based optimization, Gaussian representations significantly improve runtime while maintaining high visual fidelity. This efficiency has led to widespread adoption of Gaussian-based avatars for humans and hands, where Gaussians are initialized on canonical meshes such as SMPL [84] or FLAME [85], and animated through skeletal motion with optional non-rigid refinements [86,87,88,89,90]. Such systems demonstrate that Gaussian Splatting provides a strong balance between visual quality, editability, and computational efficiency. For interaction-centric reconstruction, BIGS [8] replaces the NeRF optimization stage with Gaussian Splatting, improving runtime and yielding an explicit representation suitable for category-agnostic object reconstruction from monocular sequences. Multi-view Gaussian frameworks such as MANUS [91] further demonstrate the potential of explicit Gaussian representations for fast reconstruction and rendering, though they require multi-view input and therefore do not generalize directly to monocular settings.

#### 4.5.4. Limitations and Emerging Directions

Despite its practical advantages, category-agnostic hand–object reconstruction remains fundamentally ill-posed under monocular occlusion. The central ambiguity lies in distinguishing hand self-occlusion from true hand–object contact occlusion when depth is only indirectly observed. While this challenge is field-wide, category-agnostic pipelines are uniquely burdened as they lack fixed object templates to resolve geometric gaps, requiring the joint inference of contact and arbitrary geometry for unseen instances [8,9].

From an optimization perspective, current systems resolve these ambiguities through coupled objective functions rather than a single geometric guarantee. Specifically, frameworks integrate image-space terms for visible regions with physical interaction constraints—such as attraction–repulsion and non-penetration penalties—to regularize the hand–object interface [8,9,91]. These are often augmented by temporal consistency and anatomical hand-model priors to reduce depth ambiguity. While such losses improve local consistency near observed contacts, they do not guarantee global identifiability under heavy occlusion, where multiple 3D configurations may satisfy the same 2D evidence.

Recent shifts toward explicit Gaussian Splatting (3DGS) pipelines, such as BIGS [8], significantly improve inference speed relative to NeRF-style optimization. However, maintaining physically stable contact reasoning over long sequences remains algorithmically challenging [8,9,83]. Consequently, the field is moving toward hybrid pipelines that combine the high-fidelity surface representation of implicit fields with the efficiency and editability of explicit Gaussians, leveraging stronger interaction-aware priors and occlusion-aware consistency constraints for more reliable reconstruction [18,57,58,92].

### 4.6. Side-by-Side Comparison

To provide a concise, side-by-side comparison across the dominant methodological families, Table 1 summarizes the typical input modalities, core characteristics, strengths/limitations, and commonly used benchmarks for model-based, CNN-, GCN-, Transformer-, and implicit/Gaussian-based pipelines. This compact taxonomy is intended as a quick reference for readers to distinguish trade-offs and evaluation resources across the field.

## 5. Hand Models

Hand models provide essential structural and geometric priors that underpin most hand reconstruction pipelines. They define how hand pose, shape, articulation, and surface deformation are parameterized, constrained, and inferred from visual observations. Over the past decade, hand modeling has evolved from simple kinematic abstractions to expressive parametric and anatomy-aware representations, enabling increasingly realistic reconstruction, interaction reasoning, and animation. In the following, we survey the predominant hand models that have guided methodological developments in hand reconstruction.

### 5.1. Kinematic Skeleton

The most fundamental representation of the hand is a tree-structured kinematic skeleton, where joints are connected by bones following predefined anatomical parent–child relationships; see Figure 1b. Such models rely on forward kinematics and joint-angle limits to enforce physically plausible poses, and have been widely used in early hand tracking systems and depth-based pose estimation [31]. Skeleton-based representations are computationally efficient and interpretable, making them suitable for real-time applications. However, they lack explicit surface geometry and cannot represent subject-specific hand shape, soft-tissue deformation, or contact-driven surface changes, which limits their realism for dense 3D reconstruction.

### 5.2. Parametric Surface Hand

To bridge the gap between pose estimation and full 3D geometry, parametric hand mesh models were introduced. The most widely adopted model in computer vision is MANO [24], which represents the hand surface using a low-dimensional parameterization of pose and shape with a fixed mesh topology. MANO follows the SMPL formulation [84], combining linear blend skinning with pose- and shape-dependent blend shapes learned from 3D scans. Its differentiability, compact parameter space, and stable animation properties have made MANO the de facto standard for monocular hand mesh recovery, two-hand reconstruction, and hand–object interaction pipelines. Despite its success, MANO models surface deformation primarily through statistical correctives and does not explicitly account for underlying anatomical structures, limiting its ability to capture muscle bulging, skin wrinkling, and contact-induced deformations.

### 5.3. Anatomy-Aware Hand

To improve physical realism, recent work has explored anatomy-inspired hand models that explicitly incorporate internal structures such as bones and muscles. PIANO [95] extends MANO by introducing anatomically correct bone geometry and joint structures derived from MRI data, enabling more realistic articulation while remaining differentiable. Building on this direction, NIMBLE [96] represents a major advance by jointly modeling bones, muscles, and skin within a unified parametric framework. NIMBLE uses rigid triangular meshes for bones and volumetric tetrahedral meshes for muscles and skin, allowing it to capture non-rigid effects such as muscle bulging and skin deformation driven by anatomically grounded constraints. By learning pose- and shape-dependent deformations from MRI supervision, NIMBLE achieves a level of realism that significantly surpasses surface-only models. Such anatomy-aware models are particularly valuable for high-fidelity animation, simulation, and data synthesis, and can also serve as differentiable priors for learning-based hand pose and shape estimation. However, their increased complexity and limited ecosystem adoption currently restrict their widespread use in large-scale vision benchmarks compared to simpler parametric models such as MANO.

### 5.4. Full-Body Models with Articulated Hands

In scenarios where hand motion must be consistent with whole-body pose, full-body parametric models are commonly employed. SMPL-X [97] extends body modeling to include articulated hands and expressive faces within a unified representation, ensuring coherent reconstruction across body parts. These models are essential in egocentric vision, avatar creation, and interaction datasets where hand pose depends on arm configuration and body motion. Nevertheless, the hand components in full-body models often inherit the limitations of surface-based hand models and typically require additional constraints or refinements to handle detailed hand–object contact and fine-grained finger articulation.

### 5.5. Hand Models in Interaction-Centric Reconstruction

For hand–object and bimanual interaction reconstruction, hand models play a central role in stabilizing pose estimation and enforcing kinematic consistency under severe occlusions. Template-based interaction approaches commonly pair MANO-like hand models with known object meshes to enforce physically plausible contact [48,73]. While such methods can achieve high geometric accuracy, they are restricted to a fixed set of object templates and struggle to generalize. More recent category-agnostic pipelines decouple hand modeling from object identity, relying on robust hand priors while reconstructing objects using implicit or Gaussian-based representations. In these systems, the hand model serves as an animatable anchor that guides occlusion reasoning, contact inference, and temporal stability during interaction reconstruction.

## 6. Discussion of Limitations and Future Trends

Despite remarkable progress in hand pose, shape, and interaction reconstruction over the past decade, the field is still far from widespread real-world adoption. From a broader perspective, the gap between benchmark performance and practical deployment is driven by accuracy limitations, computational complexity, and weak generalization in unconstrained interaction scenarios.

### 6.1. Current Limitations

Many existing methods still fail to deliver the robustness required for practical applications such as extended reality, robotics, and human–computer interaction. While recent transformer-based models, such as large-scale hand mesh recovery frameworks [57], demonstrate impressive accuracy and generalization in challenging visual conditions, they often incur substantial computational cost. High memory usage, long inference times, and reliance on heavyweight backbones limit their deployment on resource-constrained platforms, including mobile devices, wearable systems, and embedded hardware. As a result, high-accuracy hand reconstruction remains largely confined to offline workflows or high-end computing environments.

Another key limitation lies in interaction modeling. Although significant progress has been made in two-hand and hand–object reconstruction, many approaches still rely on object templates, strong supervision, or multi-view data. This restricts their scalability and applicability in open-world settings where objects are diverse, previously unseen, or partially occluded. Moreover, physically consistent contact, deformation under force, and long-term temporal stability remain challenging, particularly in monocular and egocentric scenarios.

### 6.2. Future Trends and Research Directions

Looking ahead, several converging trends are likely to shape the next generation of hand reconstruction systems. First, *category-agnostic object reconstruction* is expected to become a central component of interaction modeling. Decoupling hand understanding from object identity can enable systems to generalize across arbitrary objects and environments, significantly broadening applicability in real-world tasks.

Second, *real-time hand–object reconstruction* will become a practical requirement rather than a research luxury. Advances in efficient representations, lightweight architectures, and explicit 3D modeling paradigms are likely to bridge the performance gap between accuracy and speed, enabling deployment on consumer-grade hardware without sacrificing interaction fidelity.

Third, foundational models for hand motion understanding are likely to move beyond explicit joint coordinates toward richer *pose embeddings*, consistent with recent trends in representation learning and multimodal reasoning. To be practically useful, hand-centric pose embeddings must preserve fine-grained articulation and contact cues while remaining robust to occlusion, viewpoint changes, and domain shifts between lab datasets and in-the-wild egocentric video. Recent progress in full-human language-grounded 3D understanding, such as ChatPose and ChatHuman [98,99], suggests that retrieval- and reasoning-driven pipelines can support richer interaction with 3D pose representations. For hands, early language-aware progress is also emerging, including VLM-based interaction prediction and text-guided hand pose estimation [100,101,102]. Nevertheless, one of the most significant open research challenges remains data collection: unlike full-body settings, there is still no large-scale, standardized benchmark that jointly provides high-quality 3D hand and hand–object pose annotations together with dense, semantically grounded text descriptions of actions, intent, and contact. Recent evidence also suggests a persistent benchmark-to-reality gap: even as in-the-wild methods improve, performance remains sensitive to severe occlusion, unusual articulations, and long-tail interaction contexts that are underrepresented in curated benchmarks [58,103]. To reduce dataset bias, future benchmarks should combine controlled accuracy labels with real-world interaction coverage (egocentric views, contact-rich manipulation, demographic and environment diversity), and report cross-dataset transfer as a primary metric rather than a secondary diagnostic. Addressing this gap could enable systems that not only reconstruct hand motion, but also explain intent, describe actions, and support embodied AI with interpretable and transferable motion representations.

Fourth, *lightweight and low-supervision hand reconstruction* is receiving increasing attention as research shifts from accuracy-only benchmarks toward practical models that are efficient and less annotation-hungry. For efficiency, recent transformer systems improve robustness but remain computationally heavy (e.g., HaMeR), while lightweight baselines such as MobRecon remain important for on-device deployment [57,104]. On the low-supervision side, weakly supervised reconstruction with structured priors and uncertainty modeling shows that competitive performance is possible with mostly 2D labels, reducing dependence on expensive 3D annotations [105]. Overall, the field is converging toward deployable models that jointly optimize robustness, efficiency, and annotation cost.

Finally, the long-term vision for human–computer and human–machine interaction is a transition toward *free*, *unencumbered control*. As hand reconstruction becomes more accurate, efficient, and semantically expressive, interaction systems will increasingly rely on natural hand motion as the primary interface, reducing dependence on physical controllers, markers, or wearable sensors. This evolution will play a critical role in immersive computing, robotics, and intelligent systems that seamlessly integrate human intent and action.

## 7. Conclusions

This review surveys the evolution of hand reconstruction research from early single-hand pose estimation to modern frameworks that jointly model hand pose, hand shape, two-hand interaction, and category-agnostic object reconstruction. It highlights how advances in hand models, learning paradigms, datasets, and representations have progressively enabled richer and more realistic interaction understanding. By organizing the literature around core modeling and learning paradigms—including parametric and anatomy-aware hand models, and convolutional, graph-based, and transformer architectures, as well as neural implicit and Gaussian-based representations—this paper provides a unified perspective on how design choices affect accuracy, realism, efficiency, and generalization. Recent explicit representations such as Gaussian Splatting emerge as a promising direction, offering a practical balance among photorealism, animatability, and computational efficiency, while robust hand models continue to provide critical priors that stabilize reconstruction and interaction reasoning.

Despite substantial progress, challenges remain in achieving reliable reconstruction under severe occlusion, realistic physical contact, generalization to unseen objects, and real-time performance in unconstrained environments. Future research is expected to focus on interaction-centric, category-agnostic systems that integrate efficient explicit representations with stronger geometric and interaction priors. In parallel, integrating hand reconstruction with foundation models and multimodal models opens new opportunities for semantic reasoning and embodied artificial intelligence.

We posit that semantically aware capabilities introduced by large language and vision models (LLMs/VLMs) will not replace, but rather significantly augment, existing physics-constrained methods by providing high-level context to resolve geometric ambiguities. In this perspective, semantic reasoning contributes intent- and task-level priors, while physics-based modeling enforces contact realism, kinematic validity, and safety-critical reliability. This convergence of semantic reasoning and physical precision is expected to move the field beyond laboratory settings, enabling high-stakes practical applications such as real-time monitoring and assistance during complex surgical operations.

Overall, hand reconstruction has matured into a central problem at the intersection of vision, graphics, and human–computer interaction, with growing impact across immersive systems and intelligent interfaces.

## Figures and Tables

**Table 1:** Concise taxonomy of mainstream hand reconstruction technologies and evaluation resources.

Technology	Input Modalities	Core Characteristics	Advantages/Limitations	Typical Benchmarks
**Model-based**	Depth; RGB-D; RGB (+priors)	Parametric kinematic model; energy-based fitting; explicit constraints	**+** Plausible, interpretable, low-data. **−** Slow, init-sensitive, limited deformation	NYU [2]; ICVL [93]; BigHand2.2M [34]
**CNN-based**	Depth; RGB; RGB-D; voxels	Direct regression/heatmaps; MANO hybrids; volumetric 3D CNNs	**+** Accurate, fast, scalable. **−** Weak global reasoning; volumetric is heavy	NYU [2]; BigHand2.2M [34]; V2V [37]; FreiHAND [59]; Panoptic [94]; InterHand2.6M [60]
**GCN-based**	Image/3D features + graph	Message passing on skeleton/mesh; topology-aware refinement; hand–object coupling	**+** Topology-consistent meshes. **−** Encoder-dependent; limited global context	HO3D [39]; DexYCB [13]; ContactPose [41]; H2O [14]
**Transformer-based**	RGB; multi-view (opt.); large corpora	Self-attention for global context; token decode to pose/mesh; graph-formers add topology	**+** Occlusion-robust, strong generalization. **−** Data/compute hungry; higher latency	InterHand2.6M [60]; FreiHAND [59]; HO3D [39]; ARCTIC [75]
**Implicit & Gaussian**	RGB video; multi-view; RGB-D seq.	Per-sequence optimization of fields/3D Gaussians; category-agnostic objects	**+** High-fidelity, category-agnostic. **−** Slow optimize; occlusion/contact issues	ARCTIC [9,75]; HO3D [39];

## Data Availability

No new data were created or analyzed in this study. Data sharing is not applicable to this article.
